# Study on the Meso-Statistical Damage Constitutive Model of Coral Aggregate Seawater Concrete Incorporating Natural Aggregate Replacement Ratio Effects

**DOI:** 10.3390/ma19173753

**Published:** 2026-09-03

**Authors:** Yunfei Xie, Fuan Li, Chenyang Yuan, Weifeng Bai, Junfeng Guan, Jing Liu, Kai Wang, Yajun Lv

**Affiliations:** 1 School of Human Settlements, North China University of Water Resources and Electric Power, Zhengzhou 450046, China; xieyunfei@ncwu.edu.cn (Y.X.); lvyajun@ncwu.edu.cn (Y.L.); 2 School of Water Conservancy, North China University of Water Resources and Electric Power, Zhengzhou 450046, China; 19555726081@163.com (F.L.); junfengguan@ncwu.edu.cn (J.G.); 3 Yantai Laolan Reservoir Resettlement Service Center, Yantai 264003, China; liujsmile@126.com; 4 Yantai Architectural Design and Research Co., Ltd., Yantai 264003, China; ytsywangkai@163.com

**Keywords:** coral aggregate seawater concrete, uniaxial compression, statistical damage theory, meso-characteristic parameters, damage evolution process

## Abstract

Coral aggregate seawater concrete (CASC) capitalizes on locally sourced aggregates in marine and reef engineering, enabling in situ material utilization and conferring marked benefits in curbing conventional resource consumption and construction expenditures—a combination that underpins its considerable promise for reef infrastructure development. To date, research efforts have largely been confined to macroscopic mechanical characterization and qualitative microstructural inspections, and quantitative assessments of mesoscopic damage evolution across the full loading-to-failure process remain relatively scarce. In response, to quantitatively characterize the mesoscopic damage evolution mechanism of CASC with different natural aggregates, the present study draws upon statistical damage theory and incorporates uniaxial compressive stress–strain responses from CASC mixtures formulated with four replacement ratios (0%, 33%, 67%, and 100%) of natural coarse and fine aggregates, thereby establishing a statistical damage constitutive model. The model is intentionally structured to decipher the intricate interplay that translates progressive mesoscopic deterioration into the eventual macroscopic mechanical signature, rather than merely describing phenomenological curves. The outcomes reveal favorable concordance between model-generated predictions and experimental measurements. Introducing natural aggregates appreciably modulates the cumulative damage trajectory at the mesoscale; with rising replacement ratios, the macroscopic mechanical performance of CASC is systematically fortified, concomitant with orderly shifts in characteristic damage indices (*ε*_a_, *ε*_b_, *ε*_h_ and *H*). Specifically, when the replacement ratio of natural fine aggregate is fixed at 0%, as the replacement ratio of natural coarse aggregate increases from 0% to 100%, the values of *ε*_a_, *ε*_b_, *ε*_h_, and *H* increase by 35.8%, 36.9%, 32.2%, and 66.5%, respectively. Additionally, both the fracture damage variable *D*_R_ and the integrated transverse strain area derived from digital image correlation (DIC) exhibit monotonic ascending trends as loading advances. Collectively, these contributions offer a theoretical foundation for performance optimization and a deeper mechanistic understanding of damage behavior in CASC.

## 1. Introduction

Driven by the enduring national agenda for maritime ascendancy, reef-related infrastructure construction has been propelled into a phase of swift expansion [1,2]. Coral aggregate seawater concrete (CASC), distinguished by its capacity to tap into local aggregate sources, curtail project costs, and bolster resource circularity [3,4], is now extensively utilized in reef projects, underscoring its strategic significance for both marine resource exploitation and national security infrastructure. As a novel seawater concrete variety that takes natural coral aggregates and seawater as its primary formulation components, CASC exhibits physical and mechanical attributes analogous to those of lightweight aggregate concrete [5,6], and has thereby become a pivotal material in island engineering. However, the intrinsic drawbacks of coral aggregates—including relatively low strength, marked brittleness, high porosity, and compromised durability and mechanical performance [7,8,9,10]—render them incapable of satisfying the strength criteria demanded by island facilities and marine protective structures, a limitation that considerably hinders the wider adoption of CASC in ocean engineering [11]. Hence, in the quest to upgrade the mechanical behavior of CASC, gaining a comprehensive grasp of its mesoscopic damage evolution process is essential for rational mix proportion design and for facilitating practical engineering applications.

The pursuit of improved macroscopic mechanical performance in CASC has long been a central theme of systematic research, with compressive strength, elastic modulus, durability, and pore architecture as the primary focal points, and has given rise to a substantial body of achievements. Wang et al. [12] adopted a modification approach employing polyvinyl alcohol (PVA) and calcium sulfoaluminate (CSA) clinker to treat coral sand, aiming to curtail chloride ion leaching and reduce porosity. Cai et al. [13] incorporated PVA fibers alone, which not only elevated the dynamic compressive strength of coral aggregate concrete but also markedly ameliorated its brittle failure mode under impact loading. Wang et al. [14] implemented strategies involving defect regulation, interface reconstruction, and transport-structure optimization, thereby converting coral aggregates into an effective component for high-durability, low-carbon marine concrete, and consequently upgrading the overall mechanical performance. To circumvent the inherent performance constraints of coral aggregates, a plethora of studies have ventured to introduce fibers, apply surface modification to aggregates, or incorporate novel cementitious systems (e.g., magnesium oxysulfate cement and alkali-activated geopolymers). Such interventions have demonstrated positive outcomes in raising compressive strength, refining pore size distribution, improving impermeability, and enhancing overall durability. Nevertheless, excessive fiber dosages tend to cause non-uniform dispersion within the matrix, and may undergo aging degradation under high ultraviolet irradiation, thereby compromising long-term mechanical stability and durability [15,16,17]; improper control of aggregate pretreatment processes may introduce aggressive species like chloride ions, heightening the risk of internal steel reinforcement corrosion and shortening the service life of structures [18,19,20]; although novel cementitious materials offer unique chemical and mechanical advantages, their complex preparation procedures, relatively high economic costs, and the need for improved compatibility with specific chemical admixtures remain challenges [21,22]. Recent inquiries have indicated that partial replacement of coral aggregates with natural aggregates can improve the performance of seawater-mixed sand concrete [23,24]. Natural aggregates, characterized by higher density, lower porosity, and better ductility, can effectively augment the strength, compactness, and crack resistance of concrete [25]. Zhang et al. [26] systematically compared the compressive failure modes, stress–strain curves, and mechanical parameters of CASC with varying replacement ratios (natural coarse aggregate NCA and natural fine aggregate NFA) and concluded that the incorporation of natural aggregates refines the matrix structure, bolsters mechanical properties, and concurrently enhances ductility and crack resistance.

The elucidation of microstructural responses and damage progression, as influenced by diverse modification strategies, is pivotal to deciphering failure mechanisms, refining mechanical performance, and elevating the engineering viability of CASC. Current investigative approaches predominantly encompass industrial CT scanning [27], digital image correlation (DIC) [27,28], acoustic emission (AE) [27,29], refined mechanical testing [30], and finite element analysis (FEA) [31], all of which are oriented toward characterizing damage accumulation and the evolution of crack initiation and propagation at the micro- and mesoscales. Tian et al. [32] performed DIC-based real-time strain monitoring, through which they systematically delineated the failure behavior and energy dissipation characteristics of CAC subjected to impact compression and impact splitting tension. He et al. [33] adopted CT scanning in conjunction with volumetric deformation and mortar density as diagnostic metrics, thereby examining the deteriorating effects of various corrosive solutions on CSC. Zhang et al. [26] combined DIC with scanning electron microscopy (SEM) to probe the influence of varying natural aggregate replacement levels on the mechanical responses of CASC. Collectively, these research endeavors substantiate that microscopic characterization techniques can offer qualitative yet effective insights into the damage evolution process of CASC.

Partial replacement of coral aggregates with natural counterparts contributes to the refinement of the matrix structure and the enhancement of mechanical performance in CASC, thereby paving the way for its broader adoption in reef engineering. Nevertheless, existing studies have largely concentrated on variations in macroscopic strength indices and qualitative microscopic analyses under different replacement ratios, while quantitative investigations into mesoscopic damage mechanisms remain noticeably insufficient. Concrete, as a typical quasi-brittle material, inherently experiences a continuous damage progression in which initial defects—such as micropores and microcracks—successively nucleate, initiate, propagate, and eventually coalesce during deformation and failure; this failure behavior involves cross-scale interactions among the disordered and heterogeneous structures at the macro-, meso-, and micro-scales [34,35]. The macroscopic nonlinear stress–strain response is governed jointly by microstructural heterogeneity and the nonlinear damage evolution taking place at the microscopic level. Consequently, a systematic exploration of the mesoscopic damage evolution of CASC under varying replacement ratios of natural coarse and fine aggregates holds substantial theoretical and practical relevance for a deeper comprehension of the intrinsic physical essence underlying its damage and failure.

A potent tool for deciphering mesoscopic damage in quasi-brittle materials such as concrete is afforded by statistical damage mechanics. The parallel bar model (PBS), intended for simulating uniaxial tensile behavior of concrete, was pioneered by Krajcinovic et al. [36] back in 1982; since then, the statistical damage theory has been increasingly embraced in the damage mechanics of concrete, rock, and analogous materials. Bai et al. [37,38,39,40,41] formulated a mesoscopic statistical damage model wherein the representative volume element is conceptualized as a collection of numerous mesoscopic springs or micro-bars governed by prescribed probability distributions (e.g., Weibull and normal distributions). By deriving the statistical evolution equations that dictate the failure of these micro-elements, they successfully correlated the underlying mesoscopic damage mechanisms with the observed macroscopic nonlinear stress–strain response. The inclusion of natural aggregates refines the matrix architecture of CASC, thereby altering the initiation patterns and propagation trajectories of microcracks during uniaxial compression, and consequently exerting a regulatory influence on the progression of both fracture and yield damage at the mesoscale. Consequently, exploiting the mesoscopic statistical damage framework permits a more profound elucidation of how varying replacement ratios of natural aggregates affect the mechanical behavior of CASC.

To quantitatively analyze the mesoscopic damage evolution mechanism of CASC, this paper employs the uniaxial compressive stress–strain data at various natural aggregate replacement ratios [26] and, grounded in mesoscopic statistical damage theory, proposes a statistical damage constitutive model that explicitly accounts for the effects of both natural coarse and fine aggregate replacement levels. Proceeding from this model, we systematically chart the replacement-ratio-dependent trajectories of mesoscopic characteristic parameters (*ε*_a_, *ε*_b_, *ε*_h_ and *H*), the evolution factor *E*_v_, and the fracture damage variable *D*_R_, while simultaneously generating corresponding damage evolution portraits. Regression analysis is performed to obtain the relationships between the meso-parameters and the replacement ratio, and to quantitatively assess the variations in material toughness, strain ratio and stress ratio of peak stress state and critical stress state as functions of the natural aggregate replacement ratio. A primary objective is to unravel the mesoscopic damage evolution mechanisms and, in so doing, to bridge the gap between the macroscopic nonlinear mechanical response and the mesoscale damage progression. Tensile stresses and strains are assigned positive signs, and compressive strains are negative.

## 2. Statistical Damage Theory of Concrete

### 2.1. Theory of Intrinsic Mechanical Performance Development Mechanism

**Figure 1:**
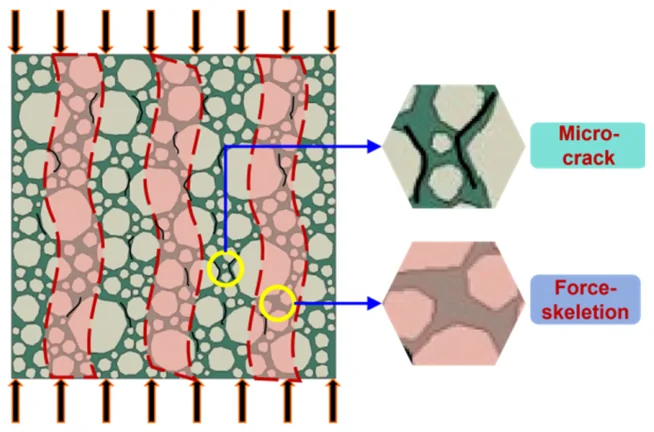
“Deterioration” and “Strengthening” of microstructure.

Yuan et al. [42] and Bai et al. [43,44] advanced a theory of “intrinsic mechanical performance mobilization,” contending that the failure and deformation behavior of quasi-brittle solids follows an evolutionary pattern analogous to that observed in ecological systems. This process can be interpreted as an adaptive self-optimization of the material’s internal architecture, driven by externally imposed loading stresses, through a “survival-of-the-fittest” selection mechanism—whereby microcrack nucleation and propagation enable weak zones to progressively shed their load-bearing function while stronger domains increasingly assume the transferred external forces, ultimately leading to a refined microstructural load-transferring framework and an enhanced capacity to accommodate the varying external mechanical environment. The interactive effects arising from this microstructural reconfiguration are summarized into two categories (see Figure 1):

(i) Degradation effect—manifested as the initiation, nucleation, and propagation of microcracks, together with the release of energy in the form of acoustic emissions.
(ii) Strengthening effect—reflected in the redistribution of internal stresses, the mutual interactions among propagating microcracks, and the accommodative adjustment of the load-bearing skeletal network.

### 2.2. Statistical Damage Constitutive Model

**Figure 2:**
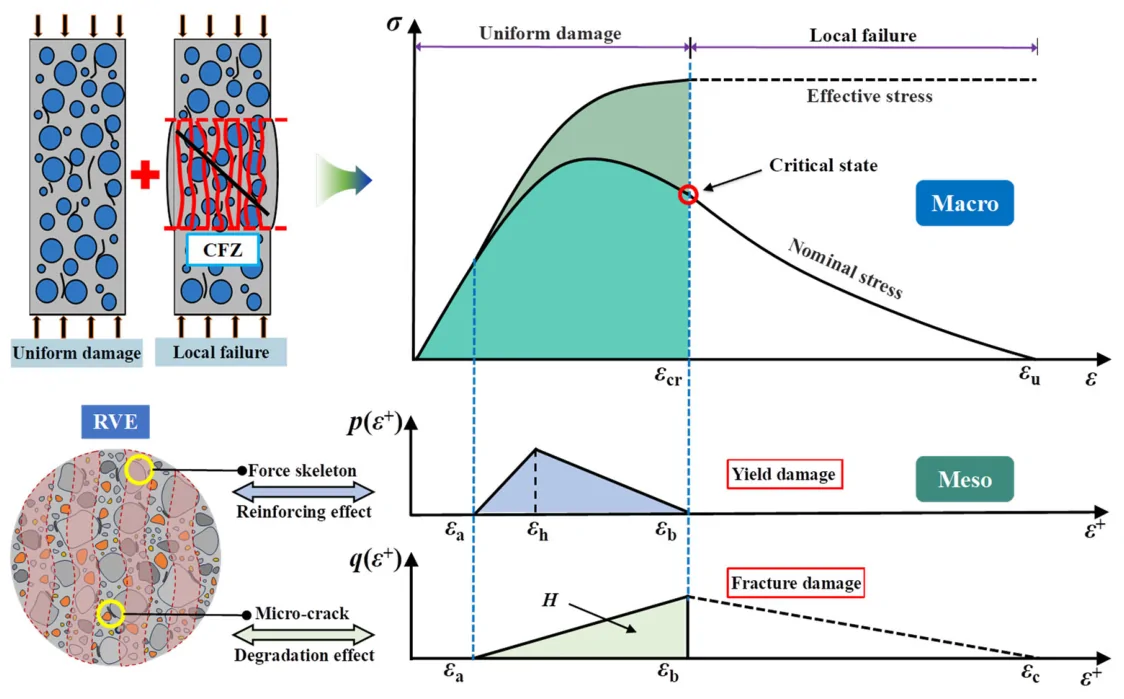
Statistical damage constitutive model under uniaxial compression.

The degradation mechanism underlying uniaxial compressive failure of concrete originates primarily from lateral tensile strain damage induced by the Poisson effect. Bai et al. [37,38,39,40,41,42,43] developed a statistical damage constitutive model (Figure 2) that incorporates an equivalent tensile damage strain *ε* + (*ε*+ > 0), defined by *ε*+ = −*νε*, with ν denoting Poisson’s ratio. This model partitions the uniaxial compression process into two sequential phases—uniform damage and localized failure—and the juncture between them is termed the critical state. The evolution of nominal stress and effective stress across these two phases is schematically portrayed in Figure 2. Within the uniform-damage phase, the nominal stress traces a rising-then-falling trajectory, whereas the effective stress climbs monotonically. Upon arrival at the critical state, the effective stress attains its peak, after which the specimen transitions into the localized-failure stage. Once this stage is entered, damage within the compressive failure zone (CFZ) intensifies progressively, eventually culminating in the formation of a diagonal shear crack band. The macroscopic nonlinear stress–strain behavior arises as a synergistic outcome of two mesoscopic damage modes—fracture and yielding—where fracture damage manifests the propagation of microcracks, while yielding damage reflects the accommodative restructuring of the microstructural load-bearing framework, corresponding in turn to the aforementioned degradation and strengthening effects. Let *q*(*ε*^+^) and *p*(*ε*^+^) denote the simplified probability density functions associated with mesoscopic fracture and yielding damage, respectively. In practical situations, *q*(*ε*^+^) and *p*(*ε*^+^) may follow sophisticated statistical laws such as Weibull or normal distributions. Given the complexity of this problem, both functions are assumed to follow a simplified triangular distribution, which can reasonably capture the major features of real statistical distributions [37]. The constitutive relation governing the uniform-damage stage is formulated as follows:

(1) $$\sigma \;={\;E}_{0}\left(1\;-\;D_{y}\right)\left(1-\;D_{R}\right)\varepsilon$$

(2) $$\sigma_{E}=E_{0}\left(1\;-D_{y}\right)\varepsilon$$

(3) $$D_{y}=\int_{0}^{\varepsilon^{+}}p\left(\varepsilon^{+}\right)d\varepsilon^{+}-\frac{\int_{0}^{\varepsilon^{+}}p\left(\varepsilon^{+}\right)\varepsilon^{+}d\varepsilon^{+}}{\varepsilon^{+}}$$

(4) $$D_{R}=\int_{0}^{\varepsilon^{+}}q\left(\varepsilon^{+}\right)d\varepsilon^{+}$$

(5) $$E_{v}=\int_{0}^{\varepsilon^{+}}p\left(\varepsilon^{+}\right)d\varepsilon^{+}$$

where σ and $\sigma_{E}$ as nominal and effective stresses, and *E*_0_ is the elastic modulus. The parameters *D*_y_ and *D*_R_ correspond to yield and fracture damage, respectively, while *E*_v_—ranging from 0 to 1—serves as an evolution factor that quantifies the extent to which the material’s latent capacity is activated. A value of *E*_v_ = 1 signals the critical state at which the latent performance reaches its fullest expression. Within the adopted formulation, *D*_y_ represents the influence of yield damage on the evolution of the effective mechanical response.

The progressive accumulation of fracture and yield damage in concrete is governed jointly by *q*(*ε*+) and *p*(*ε*+). It should be noted that the triangular distribution is a simplified approximation of mesoscopic damage probability density functions. While the major qualitative trends derived in this study provide useful insight within the current statistical framework, the numerical values and quantitative interpretation of fitted characteristic parameters depend on the assumed probability distribution. This distribution has been demonstrated to adequately characterize mesoscopic heterogeneous damage evolution [44,45]. The simplified expressions for the probability density functions *q*(*ε*+) and *p*(*ε*+) are given below:

(6) $$p\left(\varepsilon^{+}\right)=\left\{\begin{array}{cc} 0 & \left(\varepsilon^{+}\leq \varepsilon_{a}\right)\\ \frac{2\left(\varepsilon^{+}-\varepsilon_{a}\right)}{\left(\varepsilon_{h}-\varepsilon_{a}\right)\left(\varepsilon_{b}-\varepsilon_{a}\right)} & \left(\varepsilon_{a}<\varepsilon^{+}\leq \varepsilon_{h}\right)\\ \frac{2\left(\varepsilon_{b}-\varepsilon^{+}\right)}{\left(\varepsilon_{b}-\varepsilon_{h}\right)\left(\varepsilon_{b}-\varepsilon_{a}\right)} & \left(\varepsilon_{h}<\varepsilon^{+}\leq \varepsilon_{b}\right) \end{array}\right.$$

(7) $$q\left(\varepsilon^{+}\right)=\left\{\begin{array}{cc} 0 & \left(\varepsilon^{+}\leq \varepsilon_{a}\right)\\ \frac{2H\left(\varepsilon^{+}-\varepsilon_{a}\right)}{{\left(\varepsilon_{b}-\varepsilon_{a}\right)}^{2}} & \left(\varepsilon_{a}<\varepsilon^{+}\leq \varepsilon_{b}\right) \end{array}\right.$$

(8) $$H=D_{R}\left(\varepsilon_{b}\right)$$

where *ε*_a_, *ε*_b_, and *ε*_h_ are the initial damage strain, the strain corresponding to a critical state, and the peak strain of *p*(*ε*^+^), respectively. *H* is the fracture damage value corresponding to the critical state.

This model offers three principal advantages. First, it effectively captures the two-stage failure process, encompassing both the phase of distributed damage accumulation and the subsequent transition to localized catastrophic rupture. Second, within the uniform damage stage, it enables a continuous description of the damage evolution from a progressive “quantitative” accumulation to a critical “qualitative” transition. Third, and most notably, it provides a compelling mechanistic explanation for the well-documented lag of the critical state relative to the peak nominal stress—as observed in deformation localization [41], acoustic emission [46], and electrical resistivity [47] experiments. Specifically, the critical state corresponds to the attainment of the maximum effective stress, which, notably, occurs after the nominal stress peak. Once this critical point is reached, the stress skeleton within the microstructure is optimally adjusted, the material’s inherent mechanical capacity is fully mobilized, and the specimen inevitably enters the localized failure stage.

## 3. Macroscopic Mechanical Properties of Natural Aggregate Mixed Coral Aggregate Concrete

### 3.1. Complete Stress–Strain Curves

**Figure 3:**
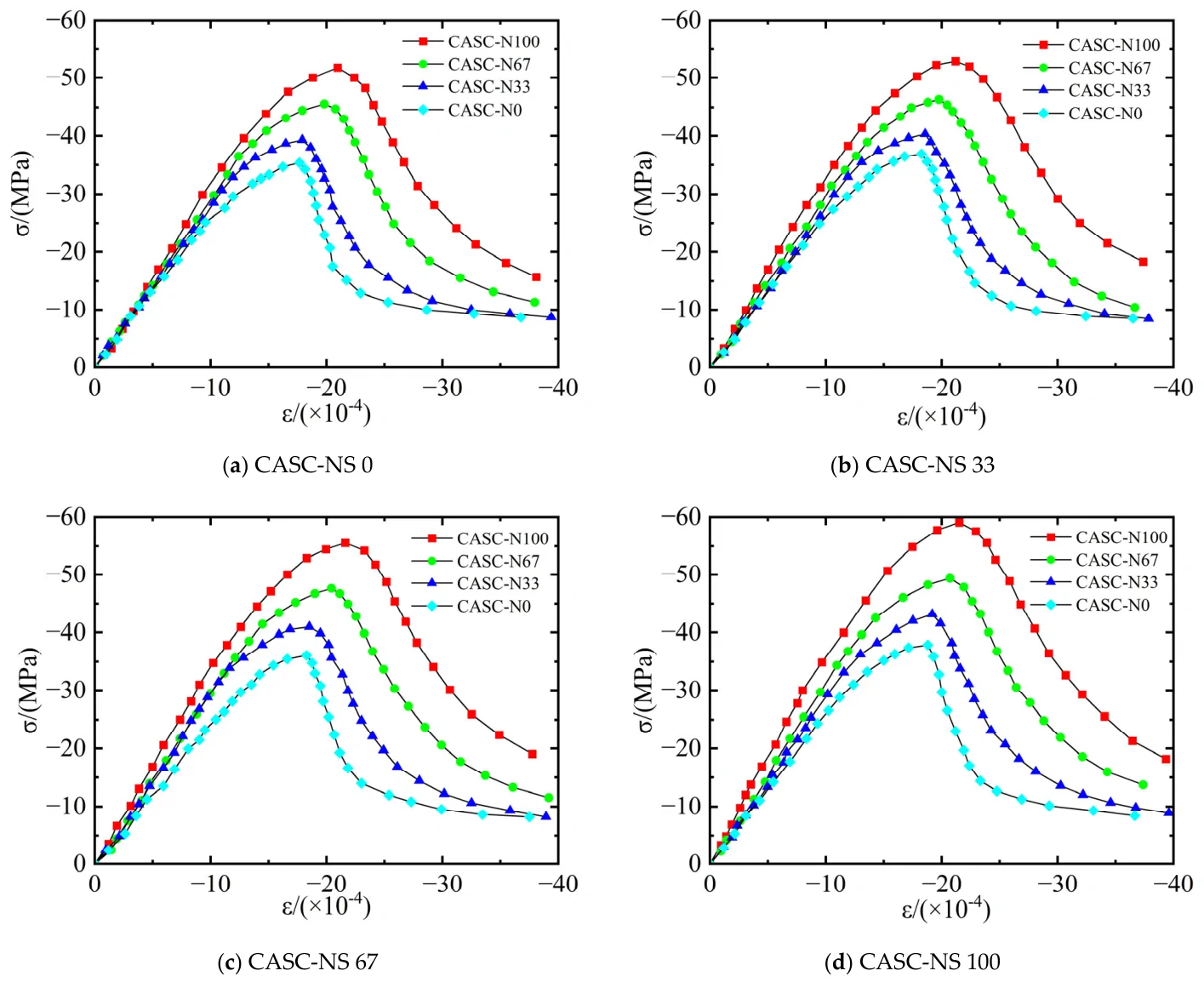
Stress–strain curves of CASC under different replacement rates of natural aggregates [26]. Note: CASC-NS33 denotes the mix proportion scheme with a 33% natural fine aggregate (NFA) replacement ratio; CASC-N33 denotes the scheme with a 33% natural coarse aggregate (NCA) replacement ratio, while CASC-NS33-N33 denotes the combined mix proportion scheme with both natural fine aggregate and coarse aggregate at 33% replacement ratios. Other abbreviations follow the same rules.

CASC specimens were prepared with seawater, cement, coral sand (CS), and coral coarse aggregate (CCA) taken from the South China Sea, according to the reference mix proportion in [26], and a polycarboxylate-based high-performance water-reducing agent at 0.3% by mass of cement was added to improve workability. Based on this mix proportion, Zhang et al. [26] conducted systematic uniaxial compression experiments with varying replacement ratios of natural coarse and fine aggregates (0%, 33%, 67%, 100%) to explore their influence on CASC performance. In this study, based on the experimental curves from this literature, the original data were re-extracted and processed using GetData software (W2.26) and then replotted to obtain the curves presented in Figure 3. The results reveal that partial substitution of coral aggregates with natural ones markedly improves the macroscopic mechanical properties of seawater sea-sand concrete. Under constant fine aggregate replacement conditions, increasing the natural coarse aggregate replacement ratio produces a regular evolution in the uniaxial compressive stress–strain curves (Figure 3), accompanied by progressively elevated peak stress, elastic modulus, and peak strain, a gentler descending branch, and enhanced ductility. These trends are primarily influenced by the intrinsic properties of the two materials described in Reference [26]: coral aggregates, which are highly porous and low in strength, induce irregular ITZs with loosely structured C-S-H gels and nonuniform pore distribution, along with elevated levels of calcium hydroxide and Friedel’s salt, which collectively weaken CASC’s mechanical behavior; conversely, dense, low-porosity natural aggregates foster compact and uniform ITZs with cement paste and a higher C-S-H content, thereby optimizing stress transfer pathways and suppressing localized strain concentrations. Further examination indicates that natural coarse aggregates, acting as the critical load-bearing skeleton, contribute prominently to enhancements in rigidity, strength, and ductility, whereas natural fine aggregates primarily exert a filling effect that bolsters workability and homogeneity but yields relatively marginal gains in mechanical properties. It should be emphasized that the macroscopic nonlinear stress–strain response across different replacement ratios is dictated not only by the replacement level itself (which modulates the initial mechanical capacity) but also by the progressive initiation, propagation, and damage accumulation of microcracks within the concrete under compression, with the latter exerting a decisive influence on both peak stress and ultimate failure patterns.

### 3.2. Transverse Strain Field Measured by Digital Image Correlation (DIC)

The lateral strain field reliably reflects the lateral expansion, stress concentration, and crack propagation behavior of concrete under compressive loading. Zhang et al. [26] demonstrated that by conducting a comparative analysis of the evolution characteristics of the lateral strain fields in CASC specimens subjected to different natural aggregate replacement rates, the stress–strain response curve during the compressive failure process can be clearly revealed. To quantify the mesoscale damage evolution during the compression process, this study employs digital image correlation (DIC) technology to obtain data on the lateral strain field at the specimen surface; the proportion of the damaged region (the red high-strain zone) is then extracted using MATLAB (R2024a). The data are derived from the DIC test results reported in reference [26], providing independent supporting evidence for examining the consistency between the experimentally observed strain-localization evolution and the damage evolution described by the statistical damage model. The DIC test results indicate that, during the initial loading stage at low stress levels, the differences in the lateral strain fields among the various specimen groups are relatively small; however, as the applied stress gradually increases, both the CASC specimens using aggregates with finite or zero replacement rates exhibit pronounced strain concentration phenomena. It is noteworthy that, while the DIC-based assessment method can partially characterize the damage state on two-dimensional lateral surfaces, it does not yet enable a systematic elucidation of the overall damage evolution pattern throughout the entire compression process; its capability to fully describe the entire damage evolution remains limited and warrants further investigation in conjunction with statistical damage theory. Therefore, this study focuses on utilizing the damage evolution data extracted via DIC as an indirect and supplementary basis for examining the consistency of the damage evolution described by the statistical damage model.

## 4. Meso-Damage Mechanism Analysis

Based on the statistical damage constitutive model, five unknown quantities (*E*_0_, *ε*_a_, *ε*_b_, *ε*_h_ and *H*) were calibrated by performing nonlinear fitting for the CASC stress–strain curves (covering ascending and partial descending branches) shown in Figure 3 using the genetic algorithm module embedded in MATLAB. For each uniaxial tensile nominal stress–strain curve, *E*_0_ is directly extracted from experimental data, which corresponds to the secant modulus connecting the origin and the point at 0.2–0.4 times peak stress on the ascending branch. The remaining four variables (*ε*_a_, *ε*_b_, *ε*_h_ and *H*) are resolved through multivariate regression implemented via the genetic algorithm component within the MATLAB toolbox. The calibration workflow is outlined below:

(1) Establish a fitness function governed by *ε*_a_, *ε*_b_, *ε*_h_ and *H*, where the sum-of-squares error between predicted and measured nominal stress serves as the objective function for optimization.
(2) Define preliminary searching bounds for the four unknown parameters.
(3) Execute the genetic-algorithm computation to generate iterative optimum results for these parameters. The parameter searching bounds are then adjusted or further narrowed based on the output performance.
(4) Iterate the computation described in step (3) until convergent optimal parameters are achieved.

**Figure 4:**
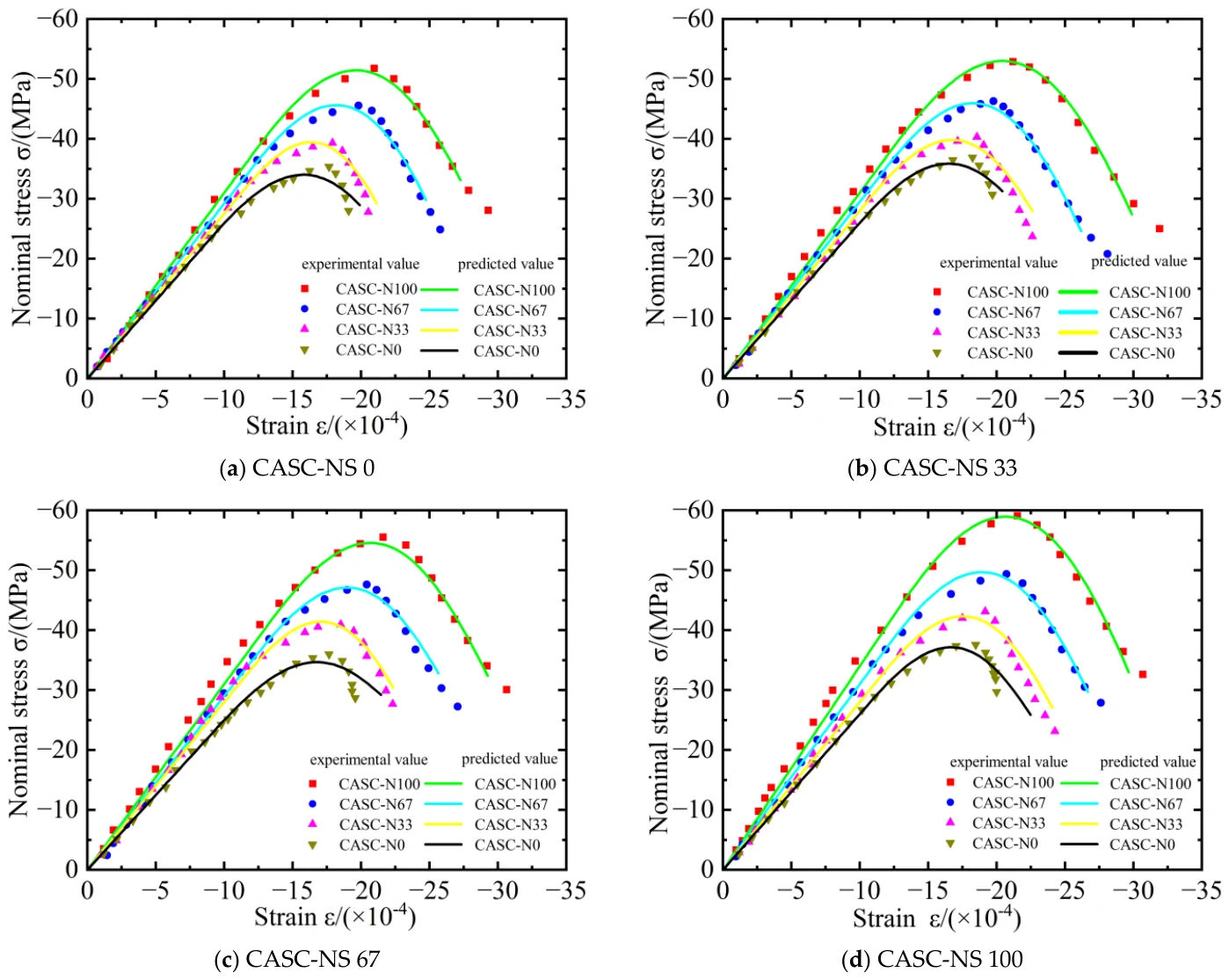
Nominal stress–strain curves.

This analysis facilitated the derivation of the requisite characteristic parameters, with the outcomes cataloged in Table 1 and Figure 4. Specifically, the parameter *R*_E_, which denotes the impact factor of the elastic modulus for coral concrete specimens under varying coarse natural aggregate substitution ratios but constant fine aggregate replacement, is mathematically defined as *E*_x%_/*E*_100%_. Within this framework, *E*_x%_ corresponds to the measured elastic modulus of concrete specimens at diverse natural coarse aggregate replacement rates (i.e., 100%, 67%, 33%, and 0%), whereas *E*_100%_ signifies the elastic modulus of the control group, serving as the baseline with a 100% natural coarse aggregate substitution level.

**Figure 5:**
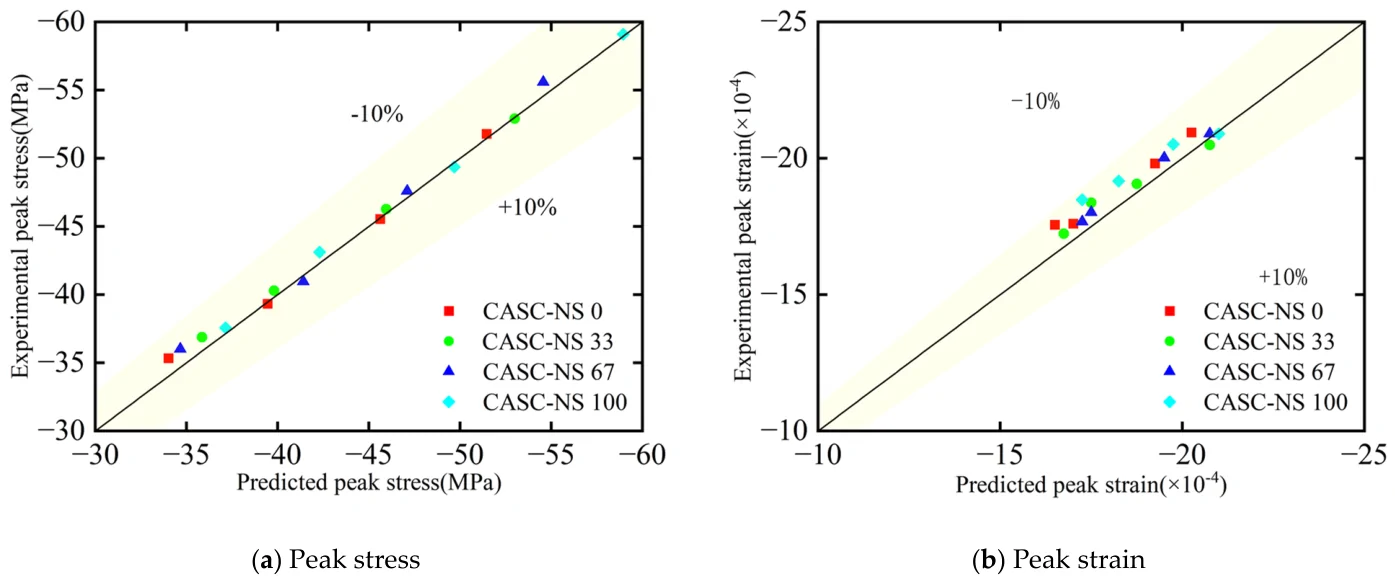
Comparison of model-predicted values and experimental values.

As illustrated in Figure 4, the fitted complete stress–strain profiles unambiguously depict two characteristic phases—namely, the ascending and descending branches—which systematically mirror the progressive accumulation and evolutionary traits of meso-damage during the whole loading-to-failure course of the specimens. A close agreement is obtained between the model-calculated curves and the experimental data used for parameter fitting, with the relative deviations in peak stress and peak strain maintained within ±10% (Figure 5). This agreement demonstrates that the proposed formulation can satisfactorily reproduce the stress–strain responses of the CASC specimens considered in the present dataset. A comparative assessment of the simulated and experimental curves across varying natural aggregate replacement levels reveals a consistent increasing tendency in both peak stress and peak strain alongside the elevated natural coarse aggregate content. This phenomenon signifies that the material’s gain in strength is concurrently coupled with a tangible amelioration in its ductility performance.

**Figure 6:**
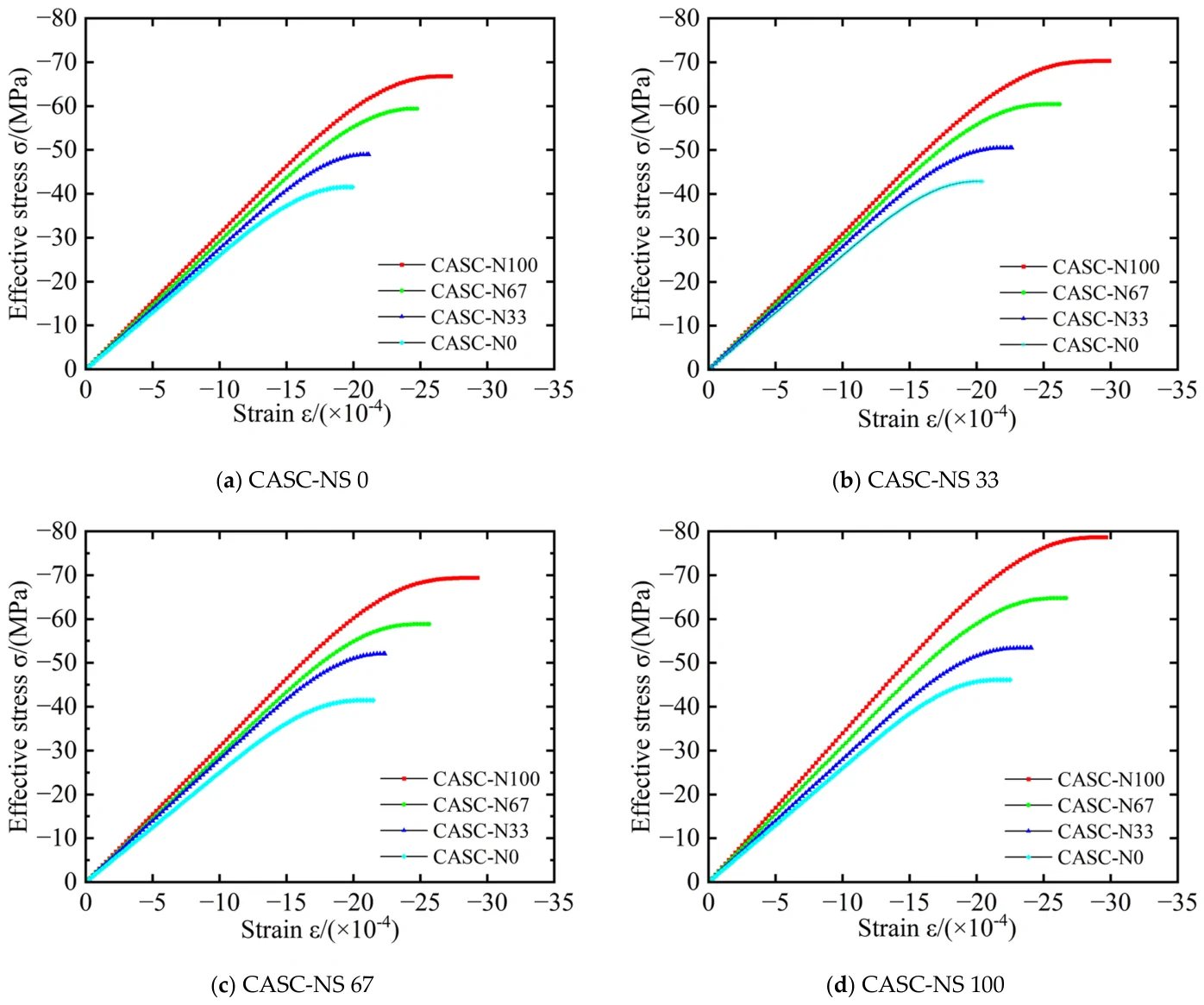
Predicted effective stress–strain curves.

Figure 6 depicts the effective stress–strain responses of CASC under varied natural aggregate replacement ratios, numerically reproduced via a numerical model rooted in the meso-statistical damage theory. During the uniform damage phase, the effective stress monotonically escalates. Upon reaching the critical threshold, the effective stress culminates at its peak, after which localized damage is initiated, driving a decline in stress and consequently marking the onset of specimen softening and structural failure. Under a fixed natural fine aggregate replacement condition, elevating the natural coarse aggregate substitution ratio yields a remarkable enhancement in both the peak effective stress and peak strain of the specimens.

### 4.1. Meso-Damage Characteristic Parameters

**Figure 7:**
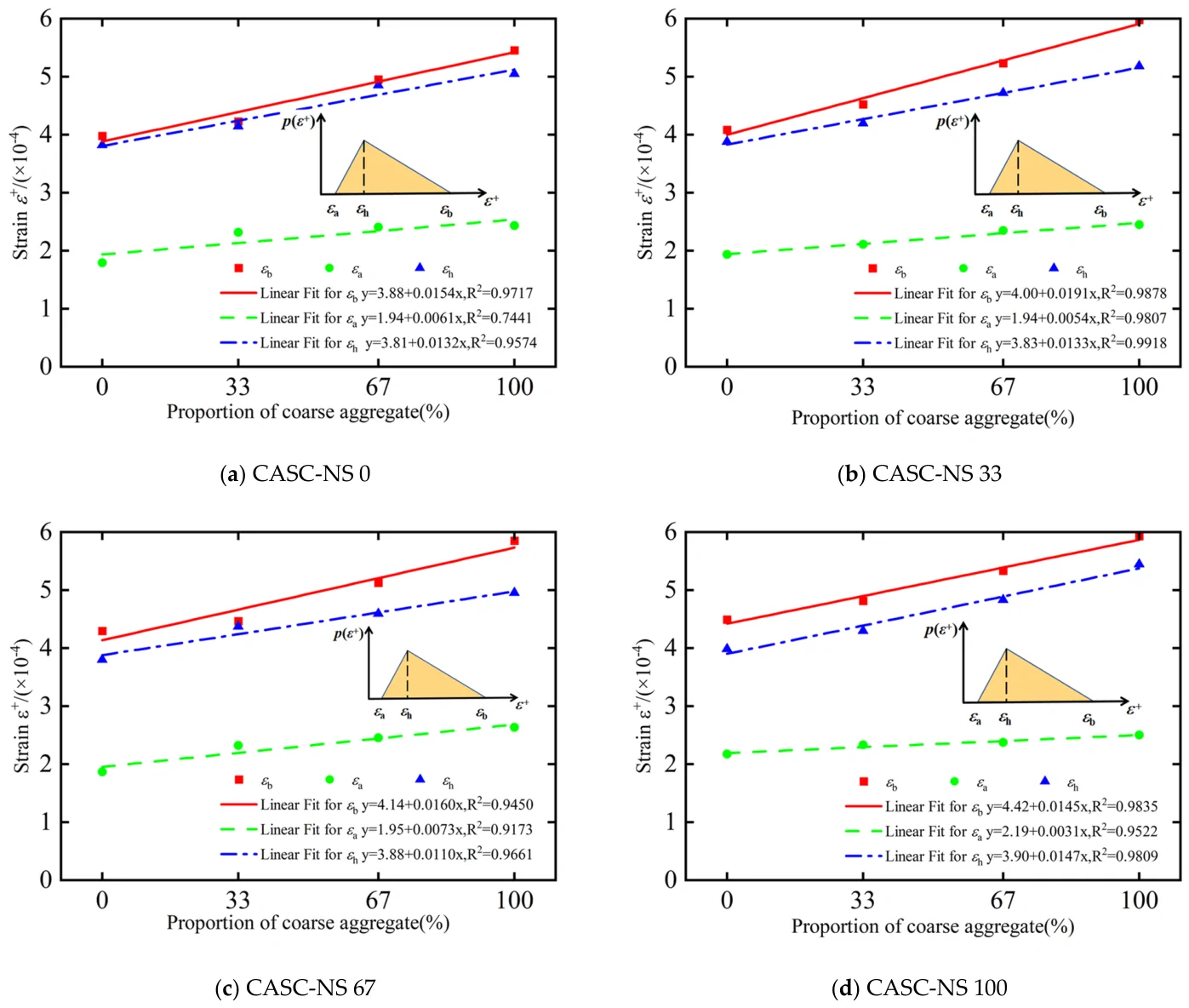
Relationships of *ε*_a_, *ε*_b_, *ε*_h_ with replacement ratio.

Figure 7 depicts the variation trends of the three meso-damage characteristic parameters (*ε*_a_, *ε*_b_, *ε*_h_) as a function of the replacement ratios of natural coarse and fine aggregates and also provides the corresponding fitting formulas. Among them, the coefficient of determination (*R*^2^) is a statistical indicator that measures the degree to which the independent variable explains the variation in the dependent variable; the closer its value is to one, the better the linear fitting effect. The average *R*^2^ value of the three meso-damage characteristic parameters is 0.9482, reflecting a good linear fitting effect. Under a constant fine aggregate substitution ratio, increasing the natural coarse aggregate content leads to an upward tendency in all three parameters. Taking the fine aggregate substitution level of 0% as an example, as the natural coarse aggregate replacement rises from 0% to 100%, *ε*_a_ increases from 1.79 × 10^−4^ to 2.43 × 10^−4^, representing a relative increment of about 35.8%; *ε*_b_ ascends from 3.98 × 10^−4^ to 5.45 × 10^−4^, with a growth rate of approximately 36.9%; and *ε*_h_ climbs from 3.82 × 10^−4^ to 5.05 × 10^−4^, recording an increase close to 32.2%. Moreover, as the fine aggregate replacement ratio rises from 0% to 100%, the increment amplitudes of these parameters also exhibit a progressively growing trend. Among these, *ε*_a_ denotes the damage initiation strain; its increase indicates that microcracks begin to nucleate only at larger strain levels. *ε*_b_ corresponds to the maximum yield damage strain at the critical state, and its enhancement reflects improved deformation capacity of the material, i.e., elevated ductility. Overall, natural aggregates at high replacement levels reinforce the skeletal structure and retard damage evolution, thereby significantly strengthening the macroscopic mechanical performance of CASC.

**Figure 8:**
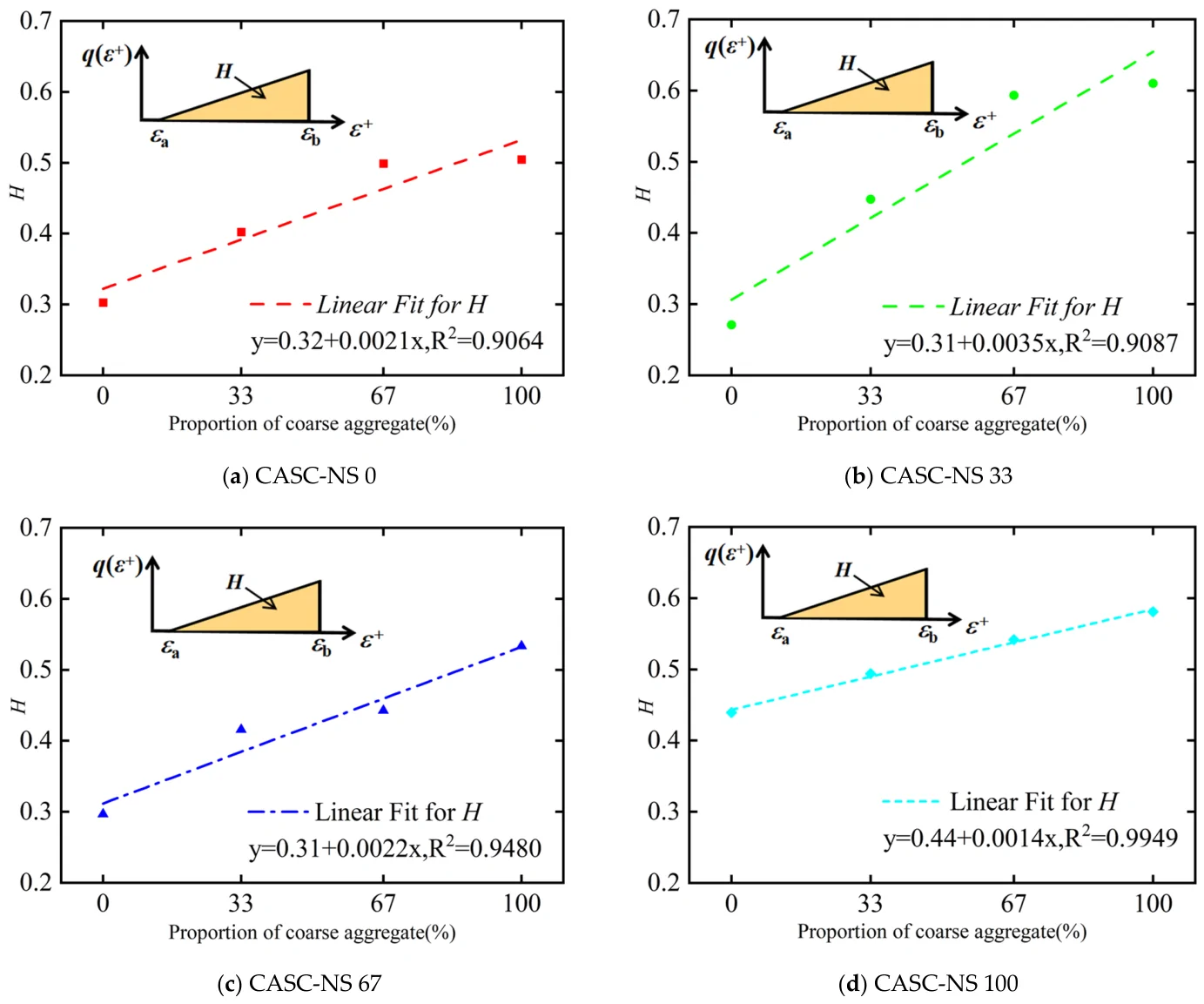
Relationship of *H* with replacement ratio.

The modulatory role of natural coarse aggregate content on the fracture-damage parameter *H* of CASC is revealed in Figure 8 for different substitution levels of natural fine aggregates, together with the associated fitting formulas. *H* can reflect the influence of increased microcrack density on the elastic modulus at the critical state, and its value is positively correlated with the increased microcrack density, but is not equal to the microcrack density itself. Under a constant fine aggregate substitution rate, *H* shows a monotonic upward trend as the coarse aggregate replacement ratio increases from 0% to 100%. When both coarse and fine aggregate replacement ratios are simultaneously elevated from 0% to 100%, *H* rises from 0.3029 to 0.5810, corresponding to an increment of approximately 91.8%. These results indicate that specimens with a higher proportion of natural aggregates undergo more pronounced adjustments in their load-bearing skeleton at the critical stage, thus exhibiting better ductility and greater potential for releasing the inherent mechanical capacity. In contrast, when coral aggregates dominate (i.e., low natural aggregate replacement), *H* remains relatively small, the critical state approaches the peak-stress state, and the post-peak curve descends steeply, marking conspicuous brittle failure characteristics.

### 4.2. Evolution Factor E_v_

**Figure 9:**
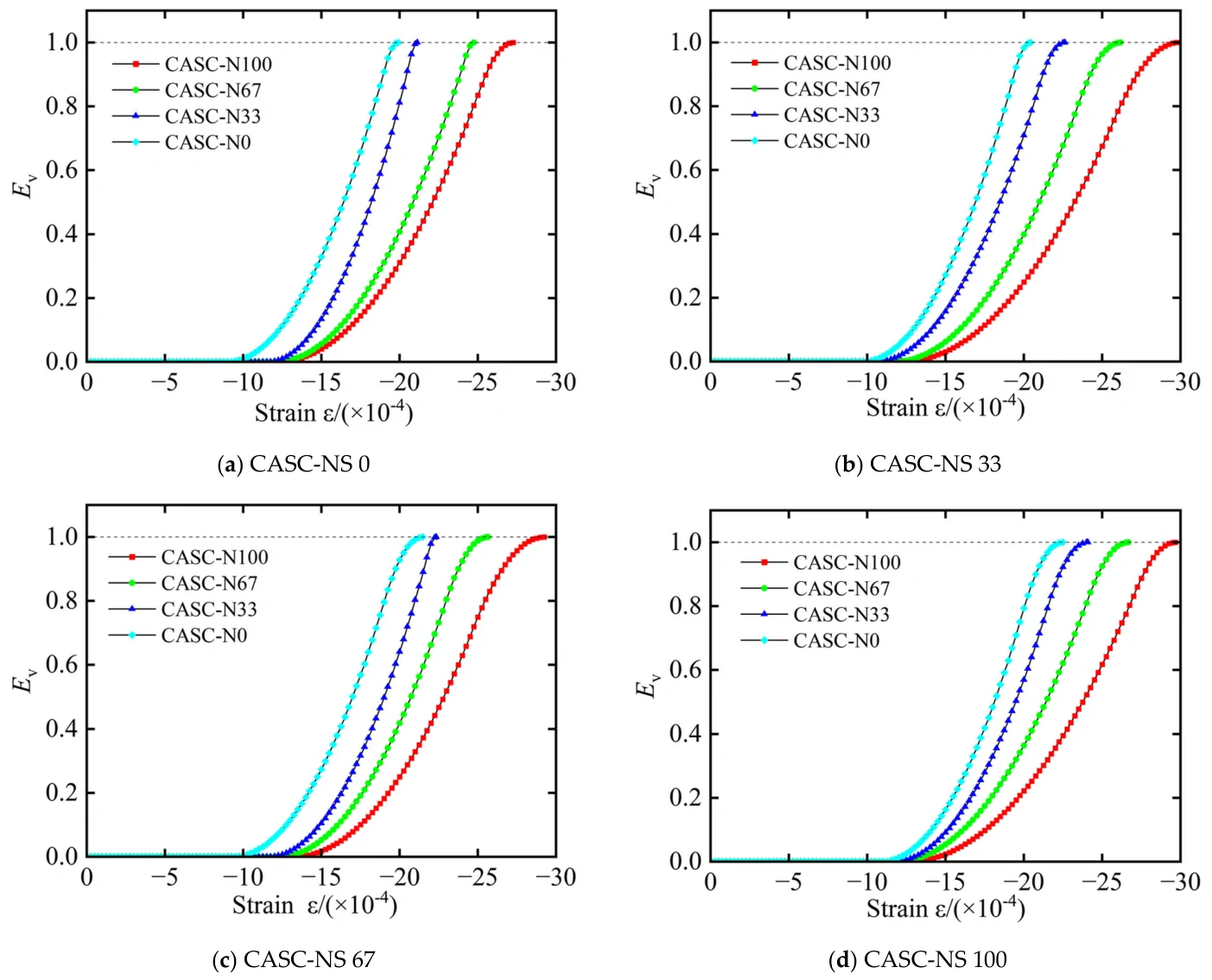
Evolution curves of *E*_v._

Figure 9 delineates the evolution patterns of factor *E*_v_ for CASC across varied natural aggregate replacement ratios. *E*_v_ is used to characterize the strengthening-related evolution represented in the statistical damage formulation. Its variation can be interpreted in terms of progressive adjustment of the internal load-bearing structure, but such structural adjustment is not directly measured in the present study. When *E*_v_ increases to one, the stress skeleton reaches the optimal critical state; deformation and damage localization [46], sudden changes in acoustic emission [48], and sudden changes in electrical resistivity [49,50] can provide experimental evidence for determining this critical state. Given a constant strain level, a lower natural coarse aggregate substitution ratio yields a larger *E*_v_ value; conversely, a higher substitution ratio induces a more pronounced rightward displacement of the *E*_v_ curve, which indicates that attaining an equivalent *E*_v_ value necessitates greater strain. This phenomenon further suggests that microcrack propagation proceeds at a more decelerated pace in high-replacement-rate specimens, thereby conferring enhanced ductile characteristics upon the material. The underlying rationale behind such behaviors is that the addition of natural coarse aggregates facilitates a more effective optimization and adjustment of the load-bearing skeleton as the loading process advances.

### 4.3. Fracture Damage Variable D_R_

**Figure 10:**
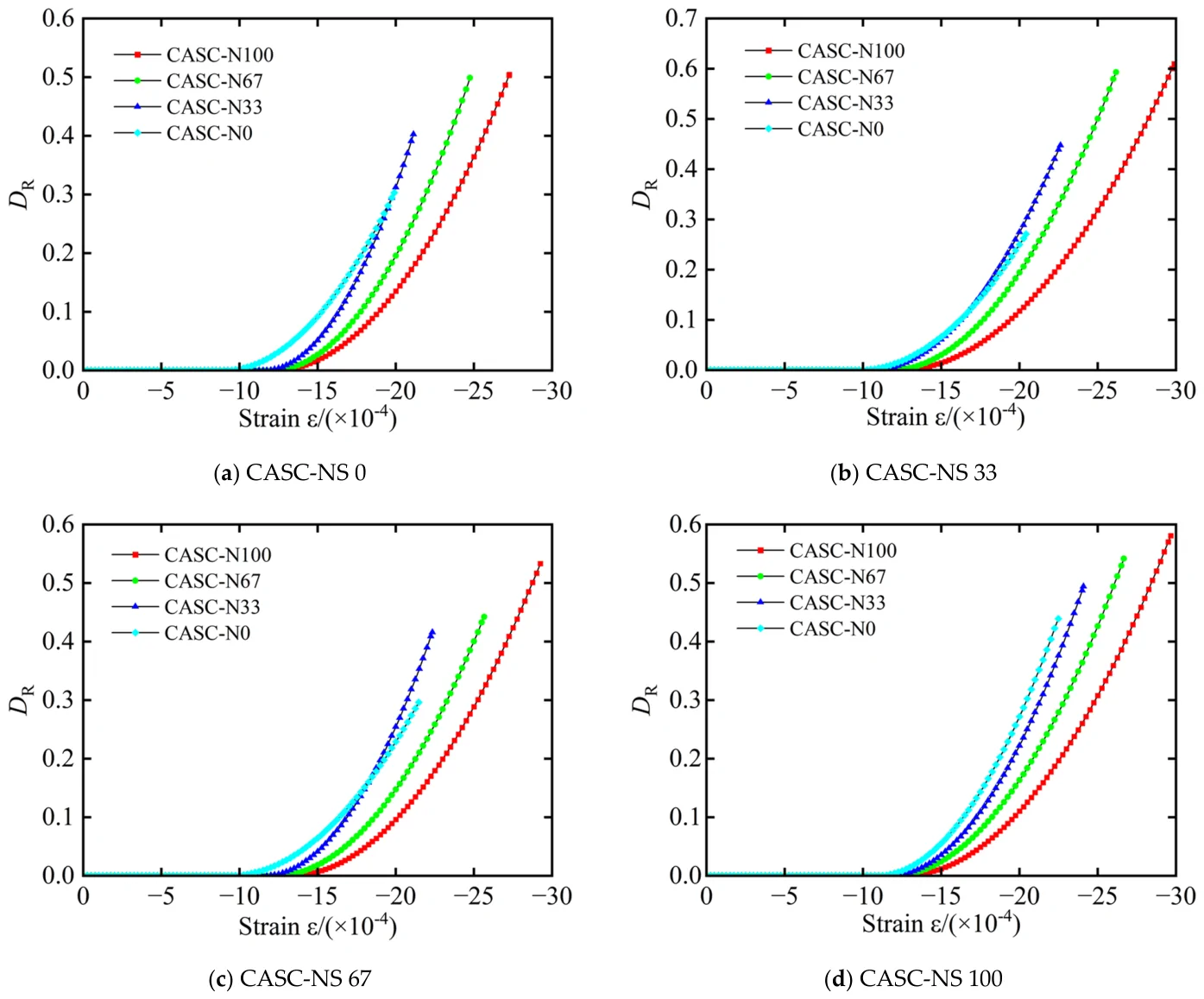
Evolution curves of *D*_R._

Figure 10 delineates the evolutionary trends of the fracture damage variable *D*_R_ for CASC with varying natural coarse aggregate replacement ratios. Within the statistical damage framework, *D*_R_ is a fracture-damage indicator associated with the cumulative evolution of fracture damage during uniaxial compression. Its variation can be interpreted in relation to the progressive development of microcracking, but it is not a directly measured quantity. *D*_R_ is equal to the area covered by the corresponding function *q*(*ε*+), and its variation trend can be qualitatively analyzed by the acoustic emission (AE) method [48]. As evidenced by the figure, with the increase in the natural aggregate replacement ratio, the initial damage strain rises, the damage accumulation tends to be more gradual, and the critical strain increases accordingly. At the macroscopic scale, as the replacement ratio increases, the peak strain increases markedly, and the slope of the post-peak descending branch decreases, collectively denoting enhanced ductility and reduced brittleness of the material. This observation corroborates that specimens with higher replacement ratios experience a more delayed onset of damage, thereby exhibiting more prominent ductile attributes. The primary rationale underpinning these phenomena lies in the fact that the inclusion of natural coarse aggregates enhances the interfacial transition zone (ITZ) between the aggregates and cement paste, rendering it denser and more homogeneous; this effect is simultaneously facilitated by a higher C-S-H gel content and a more compact microstructure. Consequently, the internal stress-transfer pathways within the concrete are effectively optimized, inhibiting the inception and extension of microcracks and substantially decelerating the overall progression of damage evolution.

### 4.4. Toughness Analysis

**Figure 11:**
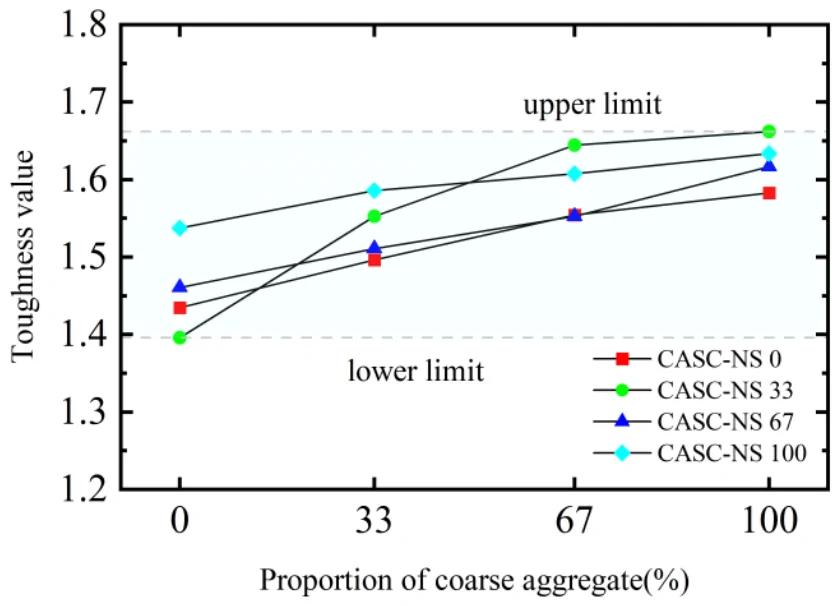
Toughness scatter plot.

Figure 11 presents the scatter distribution of the toughness ratio, which is defined as the quotient of the area beneath the fitted stress–strain curve from the damage constitutive model, integrated from zero strain up to the critical state, over that integrated up to the peak stress. With increasing coarse aggregate content, the toughness ratios across all groups exhibit a rising tendency, ranging from a lower bound of 1.396 to an upper bound of 1.662. All recorded ratios exceed unity, directly demonstrating that the strain corresponding to the critical state (including the post-peak softening branch) is larger than that at the peak stress. The marked disparity in the two sub-areas further reveals a substantial distinction between the peak state and the critical state. In addition, the elevation of natural aggregate replacement enhances the energy absorption capability of concrete, allowing the material to dissipate more energy prior to failure and thereby effectively retarding the onset of brittle fracture.

### 4.5. Comparison Between Peak Stress State and Critical Stress State

**Figure 12:**
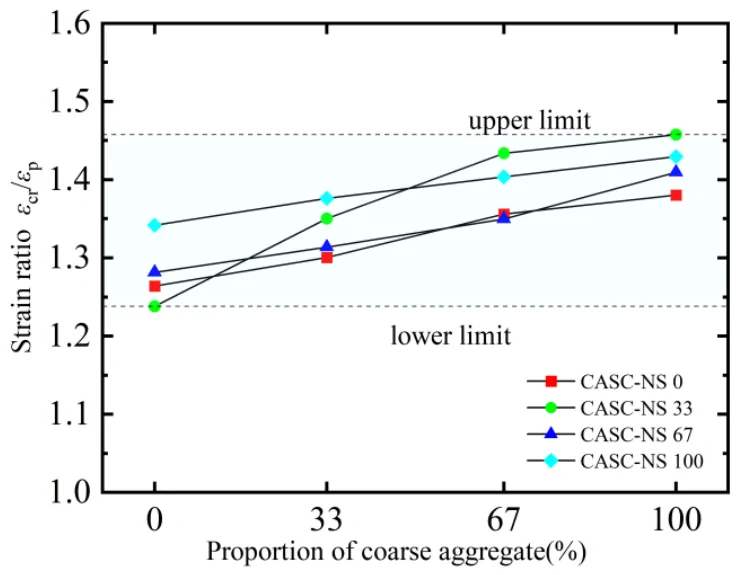
Relationship of strain (*ε*_cr_/*ε*_p_) and replacement ratio.

**Figure 13:**
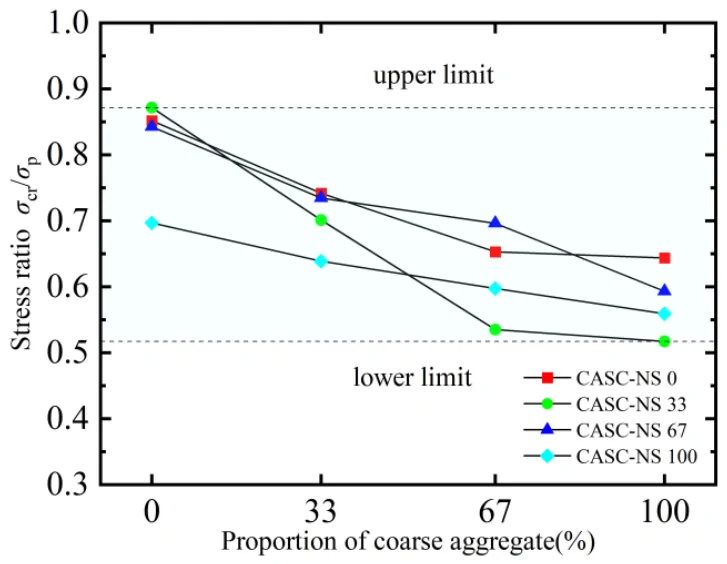
Relationship of stress (*σ*_cr_/*σ*_p_) and replacement ratio.

Figure 12 and Figure 13, respectively, present the evolutionary trends of the strain ratio (*ε*_cr_/*ε*_p_) and stress ratio (*σ*_cr_/*σ*_p_) as a function of the natural coarse aggregate replacement rate. Regarding *ε*_cr_/*ε*_p_, the upper and lower limits are observed at 1.46 and 1.24, with the average value gradually ascending from 1.28 to 1.42 as the replacement rate climbs from 0% to 100%. Conversely, the *σ*_cr_/*σ*_p_ bounds are 0.87 and 0.52, while the corresponding mean consistently decreases from 0.82 to 0.58. The observed evolutionary tendencies suggest that as the coarse aggregate replacement ratio rises, the critical state progressively shifts away from the peak stress state. Consequently, the descending branch corresponding to the uniform damage phase is prolonged, which not only enhances the ductility of CASC but also mitigates its characteristic brittle failure. To fully account for the ductile attributes of the concrete, the critical state is selected as the ultimate failure point in the current constitutive model. It is noteworthy that Xiao et al. [51] identified the state corresponding to a post-peak stress level of 0.85*σ*_p_ as the ultimate limit state, a criterion that bears a close resemblance to the definition of the critical state utilized herein.

### 4.6. Comparison Between Transverse Strain Field and Meso-Statistical Damage Analysis Results

#### 4.6.1. Transverse Strain at Different Stress States

Both the lateral strain percentage obtained by DIC technology and the fracture damage variable *D*_R_ output by the statistical damage model in this study can reflect, to a certain extent, the damage evolution of CASC under different natural aggregate replacement ratios during the loading process. Comparing the two can serve to verify the rationality of the model in capturing the macroscopic damage evolution trend. The lateral strain field serves as a reliable indicator of the lateral dilation, stress localization, and the initiation and propagation of internal microcracks within CASC under loading. As displayed in Figure 4, taking the peak stress *σ*_p_ as a benchmark, four specific stress levels—specifically 0.6σ_p_, 0.8σ_p_, 0.9σ_p_, and 1.0σ_p_—were chosen to examine the evolutionary patterns of the lateral strain field across various natural coarse and fine aggregate replacement ratios. Based on this, image processing methods were employed to quantify the data from the lateral strain nephograms at each stress level. Specifically, a program was written based on the MATLAB platform, and the RGB color thresholding method was used to define the red pixel areas in the images as high-strain/damage zones. The criterion is that the red channel intensity of the pixel must exceed 70% (R > 0.7), while the intensities of the green and blue channels must both be below 50% (G < 0.5 and B < 0.5). Only when these three conditions are simultaneously satisfied is the pixel determined as part of the damage zone. Building upon this, the area proportion of the high-strain-concentration zones (depicted in red) relative to the overall specimen surface was computed at each stress level to quantify the damage extent, with the derived outcomes cataloged in Table 2.

#### 4.6.2. Analysis of Experimental and Predicted Results

**Figure 14:**
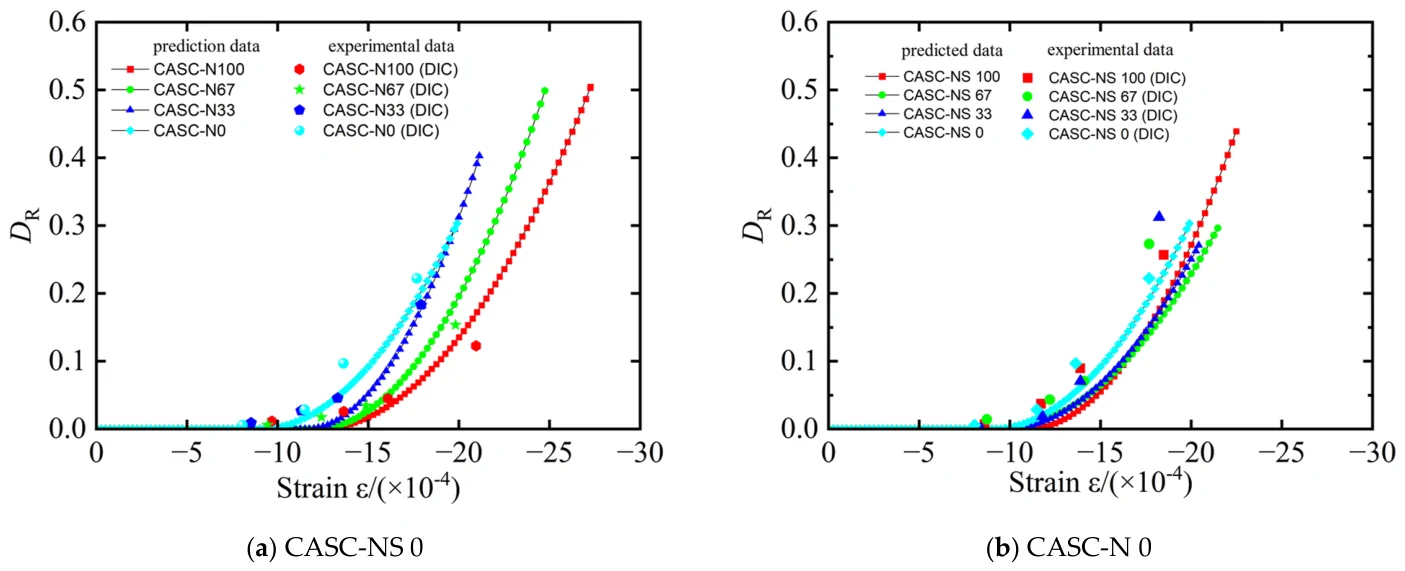
Comparative evolution curves of transverse strain percentage and DR with strain.

The fundamental distinction between DIC technology and the statistical damage constitutive model lies in their measuring dimensions: the lateral strain field primarily reflects the strain concentration and cracking information on the two-dimensional surface of the specimen, whereas the statistical damage constitutive model characterizes the evolutionary traits of microcracks throughout the entire volume. It should be pointed out that the DIC lateral strain field primarily reflects the two-dimensional surface strain localization, whereas the damage variable *D*_R_ derived from the statistical damage constitutive model represents the volumetrically averaged damage evolution within the specimen, under the assumption of the representative volume element. In other words, DR is not a directly measured three-dimensional quantity, but rather a theoretically derived indicator that characterizes the average damage state over the entire volume through the constitutive formulation. Drawing upon the data in Table 2 and the computational results presented in Figure 10, Figure 14 illustrates the interrelation between the lateral strain percentage and *D*_R_ for CASC with varying natural aggregate replacement ratios as a function of strain evolution. Both parameters exhibit a monotonically increasing trend with ascending strain; their numerical values are relatively comparable under high coarse aggregate replacement conditions, whereas a marginally expanded discrepancy emerges as the replacement rate declines. This deviation predominantly stems from the inherent differences between the two characterization methodologies—the lateral strain percentage solely reflects the damage progression on the two-dimensional surface, whereas *D*_R_ captures the overall damage evolution. Specifically, for large-sized specimens, internal damage is often more pronounced than surface damage, leading the *D*_R_ value characterizing overall damage to be higher than the DIC data reflecting surface strain; conversely, for small-sized specimens, surface damage is more likely to concentrate and manifest, causing DIC measurements to potentially surpass the model parameter *D*_R_. However, it must be pointed out that the aforementioned data quantification method based on color recognition of DIC images also has certain limitations: on the one hand, the delineation of high-strain/damage zones is highly susceptible to the influence of the operator’s subjective threshold selection; on the other hand, the criteria based on color recognition (i.e., the red regions) may not fully cover all the true physical damage inside the specimen. It should be noted that these inherent limitations are also one of the reasons why the model prediction results may differ from the measured data range in certain cases.

According to the statistical damage theory, the peak stress does not serve as the ultimate failure point for the material. Following the peak stress, microcracks continue to initiate and propagate until a critical state—denoting final failure—is eventually reached. The strain corresponding to this critical state measures approximately 1.12 to 1.38 times the peak strain, and this multiplier exhibits a positive correlation with the natural coarse aggregate replacement ratio: a higher substitution ratio corresponds to a larger multiplier, whereas a lower ratio results in a smaller value. This observation implies that specimens with high replacement levels are capable of accommodating greater deformation throughout the post-peak phase, thereby demonstrating superior ductility characteristics.

### 4.7. Damage Map

**Figure 15:**
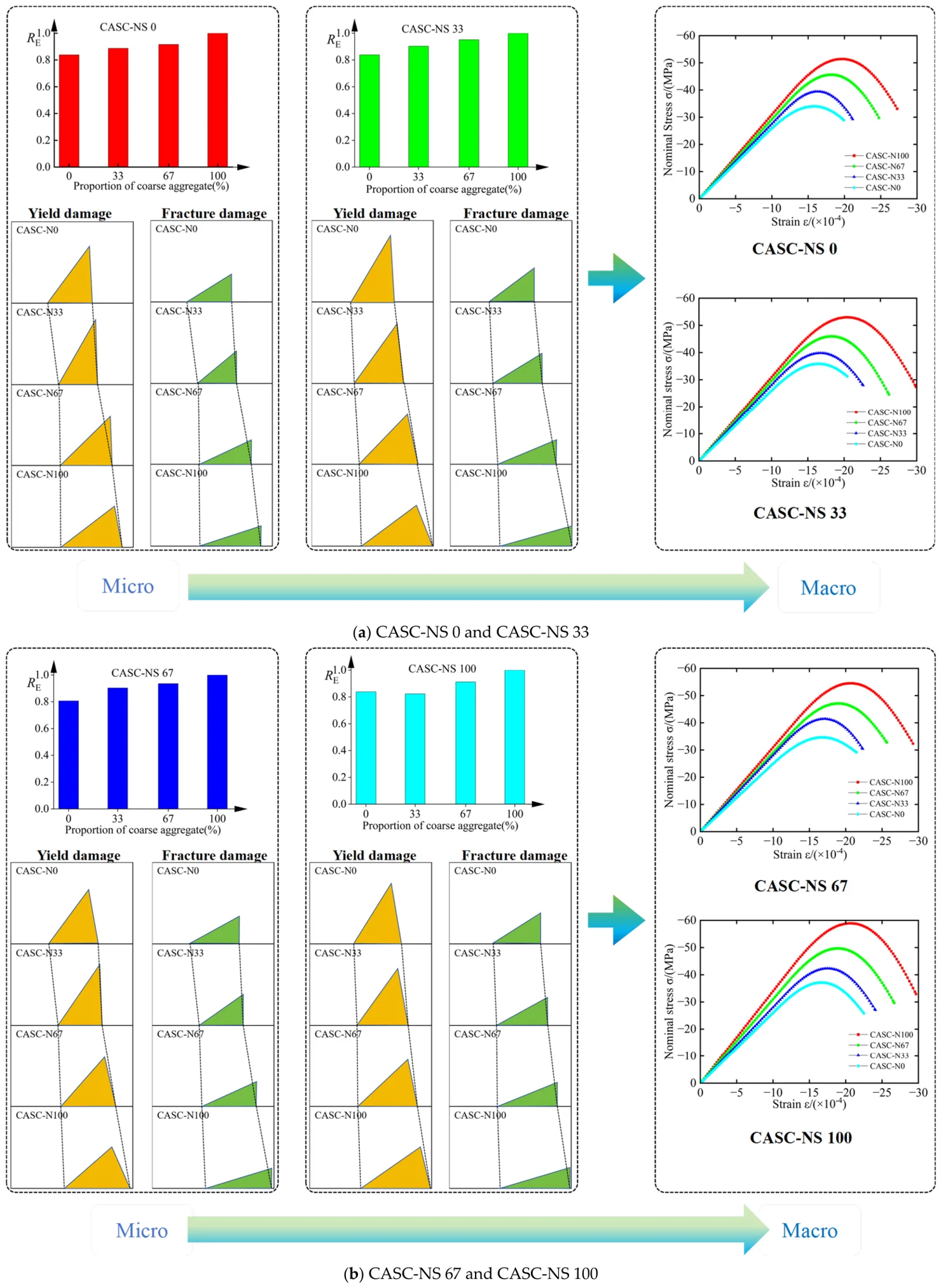
Relationship between macroscopic stress–strain behavior and meso-damage mechanism.

Figure 15 unveils the inherent linkage bridging the macroscopic nonlinear stress–strain response of CASC and its corresponding meso-damage evolution, which fundamentally involves both yielding and fracture damage mechanisms. Within this context, the elastic modulus impact factor (*R*_E_) serves to evaluate the effect of varying natural aggregate substitution levels on initial stiffness, whereas yielding damage mirrors the progressive optimization of the internal load-bearing skeleton, and fracture damage is intrinsically tied to the inception and expansion of microcracks. As corroborated by the graphical data, maintaining a constant natural fine aggregate dosage, an elevation in natural coarse aggregate content results in a distinct rightward displacement of the overall plot, accompanied by a concurrent increase in both the initial damage strain and the critical failure strain. The aforementioned alterations in meso-damage features exert a direct impact on macroscopic mechanical performances. Specifically, a consistent elevation is observed in the peak stress of the stress–strain profiles; simultaneously, the broader damage evolution span coupled with the postponement of the critical state facilitates an increment in peak strain, thereby conferring an augmented deformation capacity upon the material. These experimental findings unequivocally substantiate that, with the progressive incorporation of natural coarse aggregates, the ductility characteristics inherent to CASC become increasingly pronounced.

## 5. Conclusions

Based on the uniaxial compression stress–strain data of CASC with different natural aggregate replacement ratios, a mesoscale statistical damage constitutive model was established within the existing statistical damage framework. The model was calibrated against the available experimental stress–strain responses and subsequently used to examine the evolution of mesoscopic damage indicators. The main conclusions are as follows:

(1) The statistical damage constitutive model provides a satisfactory representation of the stress–strain responses of CASC for the experimental dataset considered in this study. The calculated peak stresses and corresponding peak strains show relative deviations within 5% and 10%, respectively, indicating satisfactory fitting accuracy. The model separates the macroscopic nonlinear response into fracture and yield-related damage components within the adopted statistical framework.
(2) Increasing the natural aggregate replacement ratio systematically changes the mesoscopic characteristic parameters. The parameters *R*_E_, *ε*_a_, *ε*_b_, *ε*_h_ and *H* generally increase, while the evolution factor *E*_v_ and fracture-damage indicator *D*_R_ exhibit a rightward shift. These results indicate that higher natural aggregate replacement is associated with delayed evolution of the damage state represented by the model and with the experimentally observed improvement in deformation capacity. The detailed physical interpretation of these quantities should, however, be understood within the adopted statistical damage framework.
(3) The critical state identified by the constitutive formulation occurs after the peak nominal stress. As the natural coarse aggregate replacement ratio increases from 0% to 100%, the average stress ratio (*σ*_cr_/*σ*_p_) decreases from 0.82 to 0.58, whereas the average strain ratio (*ε*_cr_/*ε*_p_) increases from 1.28 to 1.42. These results support the use of the critical state as the terminal state of the constitutive formulation. The fracture-damage indicator *D*_R_ and the DIC-derived lateral strain area both increase with loading, showing a consistent damage-evolution trend, although the two quantities characterize different aspects of the material response.
(4) The present analysis provides a quantitative link between the macroscopic stress–strain response and the mesoscopic damage evolution represented by the statistical damage model. The results suggest that variations in the natural aggregate replacement ratio are associated with changes in the initial mechanical response and the subsequent evolution of fracture and yield-related damage within the adopted framework. The observed DIC strain-localization characteristics provide supplementary experimental evidence for the corresponding damage-evolution trends. However, the damage variables should not be regarded as direct measurements of three-dimensional internal damage or microcrack evolution.
(5) The results provide a quantitative basis for understanding the influence of natural aggregate replacement on the mechanical response of CASC within the investigated mixture of proportions and uniaxial compression conditions. The applicability of the identified parameters and relationships to other mixture proportions, material strengths, loading conditions, and aggregate characteristics requires further verification. In addition, the potential dependence of the fitted parameters on the assumed triangular probability distributions should be considered when extending the model to other material systems.

## Figures and Tables

**Table 1:** Damage characteristic parameters.

Type	Group	*ε*_a_ × 10^−4^	*H*	*ε*_h_ × 10^−4^	*ε*_b_ × 10^−4^	*R*_E_
CASC-NS 0	CASC-N0	1.79	0.3029	3.82	3.98	0.839
	CASC-N33	2.32	0.4025	4.14	4.23	0.887
	CASC-N67	2.41	0.4988	4.85	4.95	0.916
	CASC-N100	2.43	0.5045	5.05	5.45	1
CASC-NS 33	CASC-N0	1.93	0.2711	3.88	4.09	0.839
	CASC-N33	2.11	0.4476	4.20	4.52	0.903
	CASC-N67	2.35	0.5932	4.72	5.23	0.952
	CASC-N100	2.45	0.6099	5.18	5.98	1
CASC-NS 67	CASC-N0	1.87	0.2965	3.80	4.29	0.806
	CASC-N33	2.32	0.4156	4.37	4.47	0.903
	CASC-N67	2.46	0.4425	4.59	5.13	0.935
	CASC-N100	2.63	0.5335	4.95	5.85	1
CASC-NS 100	CASC-N0	2.17	0.4393	3.98	4.49	0.839
	CASC-N33	2.33	0.4939	4.30	4.82	0.824
	CASC-N67	2.37	0.5416	4.83	5.33	0.912
	CASC-N100	2.51	0.5810	5.45	5.93	1

Note: *ε*_a_ denotes the initial damage strain; *ε*_b_ denotes the maximum yield damage strain; *ε*_h_ denotes the strain at the *p*(*ε*+) peak; *H* denotes the fracture-damage value corresponding to the critical state defined within the adopted constitutive framework; *R*_E_ denotes the influence factor of the coarse aggregate replacement ratio (with fine aggregate fixed) on the elastic modulus of coral concrete.

**Table 2:** Transverse strain percentages of CASC at different stress levels.

Type	Percentage of Lateral Strain (%)			
	0.6*σ*_p_	0.8*σ*_p_	0.9*σ*_p_	1.0*σ*_p_
CASC-NS 0-N0	0.58	2.88	9.71	22.22
CASC-NS 33-N0	0.13	1.86	7.04	31.19
CASC-NS 67-N0	1.43	4.34	7.10	27.31
CASC-NS 100-N0	0.72	3.76	8.99	25.69
CASC-NS 0-N33	0.91	2.69	4.55	18.33
CASC-NS 0-N67	0.57	1.75	3.44	15.32
CASC-NS 0-N100	1.17	2.61	4.48	12.30

## Data Availability

The original contributions presented in this study are included in the article. Further inquiries can be directed to the corresponding authors.
